# Changes in ceftriaxone pharmacokinetics/pharmacodynamics during the early phase of sepsis: a prospective, experimental study in the rat

**DOI:** 10.1186/s12967-016-1072-9

**Published:** 2016-11-15

**Authors:** Valentina Selmi, Beatrice Loriga, Luca Vitali, Martina Carlucci, Alessandro Di Filippo, Giulio Carta, Eleonora Sgambati, Lorenzo Tofani, Angelo Raffaele De Gaudio, Andrea Novelli, Chiara Adembri

**Affiliations:** 1 Department of Health Sciences, Section of Anesthesiology and Intensive Care, University of Florence, Azienda Ospedaliero-Universitaria Careggi, Largo Brambilla 3, 50134 Florence, Italy; 2 Department of Biosciences and Territory, University of Molise, Contrada Fonte Lappone, 86090 Pesche, IS Italy; 3 Department of Health Sciences, Section of Clinical Pharmacology and Oncology, University of Florence, Viale Pieraccini 6, 50139 Florence, Italy; 4 Department of Neurosciences, Psychology, Drug Research and Child Health, University of Florence, Florence, Italy

**Keywords:** Ceftriaxone, Sepsis, Pharmacokinetics/pharmacodynamics, Glomerular filtration barrier, Glycocalyx, Sialic acids, Monte Carlo simulation

## Abstract

**Background:**

Sepsis is characterized by the loss of the perm-selectivity properties of the glomerular filtration barrier (GFB) with consequent albuminuria. We examined whether the pharmacokinetics–pharmacodynamics (PK/PD) of ceftriaxone (CTX), an extensively protein-bound 3rd generation cephalosporin, is altered during early sepsis and whether an increase in urinary loss of bound-CTX, due to GFB alteration, can occur in this condition.

**Methods:**

A prospective, experimental, randomized study was carried out in adult male Sprague–Dawley rats. Sepsis was induced by cecal ligation and puncture (CLP). Rats were divided into two groups: Sham-operated and CLP. CTX (100 mg i.p., equivalent to 1 g dose in humans) was administered in order to measure plasma and lung CTX concentrations at several time-points: baseline and 1, 2, 4 and 6 h after administration. CTX was measured by High Performance Liquid Chromatography (HPLC). The morphological status of the sialic components of the GFB barrier was assessed by lectin histo-chemistry. Monte Carlo simulation was performed to calculate the probability of target attainment (PTA >90%) for 80 and 100% of T_free_ > minimum inhibitory concentration (MIC) for 80 and 100% of dosing interval.

**Measurements and main results:**

After CLP, sepsis developed in rats as documented by the growth of polymicrobial flora in the peritoneal fluid (≤1 × 10^1^ CFU in sham rats vs 5 × 10^4^–1 × 10^5^ CFU in CLP rats). CTX plasma concentrations were higher in CLP than in sham rats at 2 and 4 h after administration (difference at 2 h was 47.3, p = 0.012; difference at 4 h was 24.94, p = 0.004), while lung penetration tended to be lower. An increased urinary elimination of protein-bound CTX occurred (553 ± 689 vs 149 ± 128 mg/L, p < 0.05; % of bound/total CTX 22 ± 6 in septic rats vs 11 ± 4 in sham rats, p < 0.01) and it was associated with loss of the GFB sialic components. According to Monte Carlo simulation a PTA > 90% for 100% of the dosing interval was reached neither for sham nor CLP rats using MIC = 1 mg/L, the clinical breakpoint for *Enterobacteriacee*.

**Conclusions:**

Sepsis causes changes in the PK of CTX and an alteration in the sialic components of the GFB, with consequent loss of protein-bound CTX. Among factors that can affect drug pharmacokinetics during the early phases of sepsis, urinary loss of both free and albumin–bound antimicrobials should be considered.

## Background

The effects of sepsis on the pharmacokinetics-pharmacodynamics (PK/PD) of antimicrobials have been extensively examined in recent years [1]. A major contribution to sepsis-induced changes in PK/PD is due to modification in the volume of distribution (Vd) and renal clearance. The latter [1–3] is frequently impaired and this fact may reduce drug elimination. However, in up to 20% of intensive care unit (ICU) patients, an increase in creatinine clearance (CrCL) occurs in the initial phases of sepsis and, as a consequence, an increase in drug elimination can ensue, with reduction in pathogen exposure to antimicrobials [4]. Theoretically, the increase in CrCL should concern only the “free” antimicrobial, which is physiologically capable of crossing the glomerular filtration barrier (GFB), since the passage of ligand proteins such as albumin is normally prevented by the integrity of the GFB itself. A major contribution to the perm-selective properties of the GFB is provided by the glycocalyx, a network of glycoproteins, proteoglycans and soluble components which line the extracellular surface of all cells, including the glomerular endothelial cells and podocytes [5]. However it has been shown that sepsis impairs GFB properties, since albuminuria is an early marker of postoperative patients developing sepsis [6]. It is therefore likely that during sepsis even the quantity of antimicrobials bound to albumin is eliminated in the urine, which is consistent with the fact that the molecular weight of antimicrobials is usually negligible in comparison to albumin and other ligand proteins (<1000 vs 69,000 Da respectively) [7]. Among the antimicrobials indicated for treating abdominal or uro-sepsis in ICU patients, ceftriaxone (CTX), a highly protein-bound (>95%), third-generation cephalosporin [8], maintains a role when susceptible pathogens are involved. For CTX, a time period during which free concentrations remains higher then MIC (T_free_ > MIC) for at least 80% of the dosing interval is considered the optimal PK/PD target [9]. In critically ill patients, a fast (within 24–48 h) achievement of antimicrobial target concentrations is also of special importance in order to improve the outcome [1, 10].

Studies in ICU septic patients have shown very high variability in the main PK parameters of CTX, with changes in renal clearance representing the main cause [11]. No data is reported concerning increased elimination of CTX due to loss of GFB perm-selectivity.

In the present study, we have aimed therefore at evaluating in an experimental model of sepsis whether (1) changes in the PK of CTX occur from the beginning of sepsis, (2) whether these changes are associated with changes in the renal clearance of bound/unbound CTX and changes in the GFB perm-selectivity, (3) whether these changes have consequences on the probability of reaching the PK/PD target (calculated using Monte Carlo simulation).

## Methods

Adult male Sprague–Dawley rats (Harlan, Udine, Italy) weighing 320–330 g were used for this study. The experimental protocol was approved by the Committee for Animal Experimentation of the Ministry of Health, Rome, Italy. Animals were treated according to Italian and European Guidelines for Animal Care and Experimentation, DL 116/92, in agreement with the European Communities Council Directive guidelines (86/609/EEC).

**Fig. 1:**
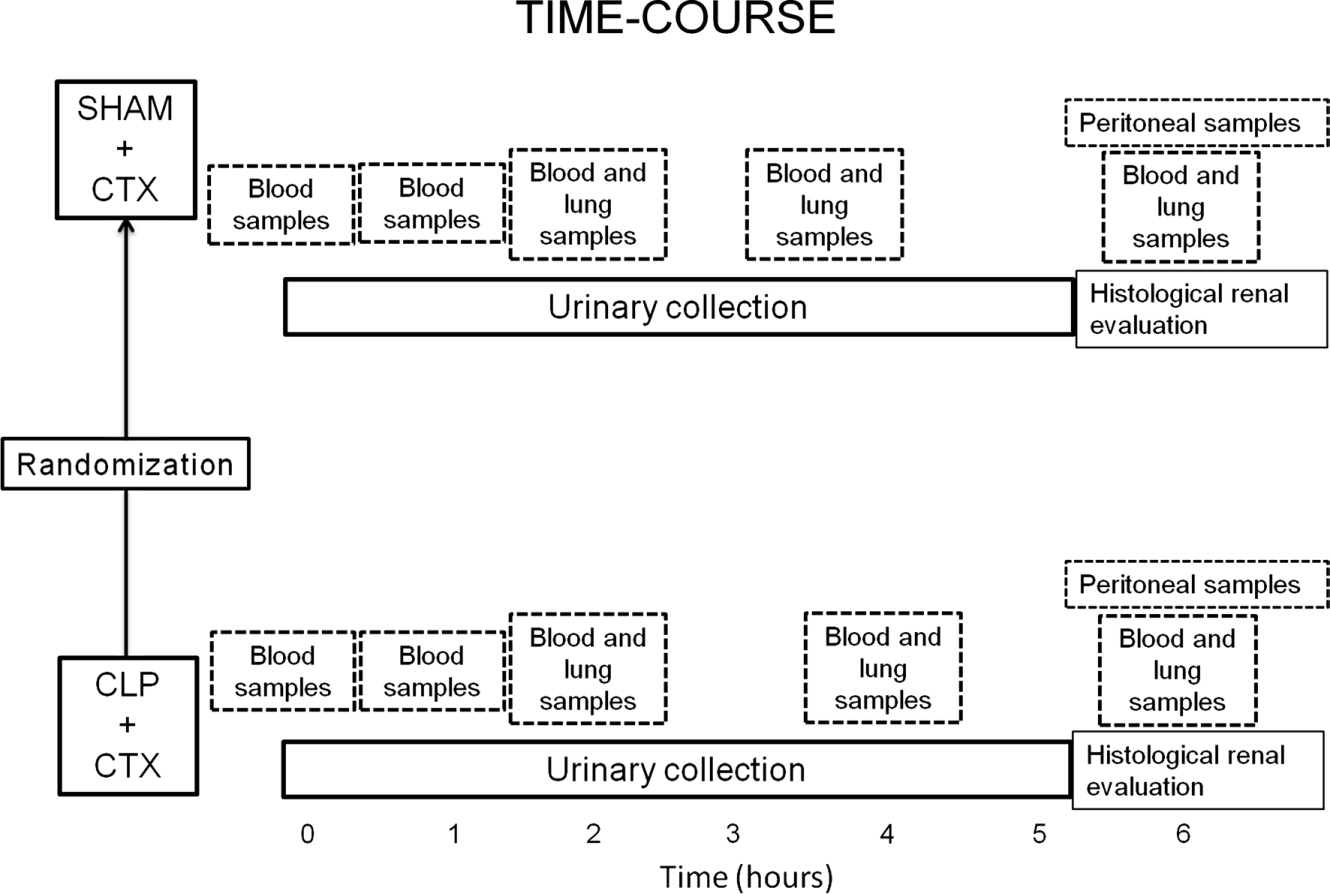
Experimental time course

The SD rats were initially housed three per cage in a temperature (18–22 °C) and humidity-controlled room under a constant 12-h light/dark cycle and fed with standard rat chow and water ad libitum. After acclimatization, animals were identified from number 1–36 and then, by using the Microsoft Excel software (Lombardia, Italy), assigned to one of the two study groups by a blind operator. Eighteen rats underwent sepsis (induced by cecal ligation and puncture (“CLP group”), and 18 rats (who received laparotomy only) served as controls (“Sham-group”). At the end of the surgical procedure each rat received the same amount of CTX intra-peritoneally (i.p.)(see below). A schematic representation of the experimental design is shown in Fig. 1.

The CLP procedure was performed as previously detailed [12–14]. Briefly, rats anesthetized with sodium pentobarbital (65 mg/kg, i.p.) and positioned on a homoeothermic heating pad in order to maintain body temperature between 36.5 and 37.5 °C, received a 3 cm midline laparotomy on the anterior abdomen. The cecum was exposed and ligated distally to the ileocecal valve, without causing intestinal obstruction, and it was punctured on the anti-mesenteric border with a 16 G (1.65 mm diameter) needle. The cecum was then squeezed to extrude its fecal content and was replaced into the peritoneal cavity; finally, a 20 G cannula was positioned in the peritoneal cavity and secured to the abdominal wall in order to subsequently inject CTX and/or collect peritoneal samples. The anterior peritoneal wall and the skin were closed with 3-0 silk sutures. “Sham” animals underwent laparotomy and peritoneal cannula positioning only. After surgery, all the rats were housed individually for urine collection in metabolic cages in the same environmental conditions.

To document the development of sepsis, the clinical appearance, mortality during the experimental time (6 h) and micro-organism growth in the peritoneal cavity were evaluated. For micro-organism detection and count, samples of peritoneal fluid were collected at the end of the experimental time (6 h) and cultured for the growth of Gram-positive and Gram-negative isolates. Samples were incubated on Mannitol Salt Agar for 24 h at 37 °C for Gram-positive strains and layered on Mac-Conkey Agar III for 24 h at 37 °C for Gram-negative culture. Species identification was determined by the API system (BioMerieux, Inc., Hazelwood, MO), using a panel Gram-positive (GP) card. The effects of sepsis on the status of the GBF, and on CTX elimination were also examined (see below).

The CTX was purchased from Sigma-Aldricht Co. (Saint Louis, Missouri, USA). The antimicrobial solution was prepared daily by dissolving the powder in sterile saline. 100 mg/kg CTX was injected intra-peritoneally through the 20 G abdominal cannula in a volume of 1 mL; the cannula was flushed immediately with 1 mL of saline. The dose of CTX was chosen according to previous experimental protocols in order to mimic the human dose of 1 g [15].

Plasma and lung concentrations were evaluated for the first 6 h after CTX administration. Serial blood samples (drawn from the tail vein) were collected at the following times: before the administration of the antibiotic (baseline sample) and after 1, 2, 4 and 6 h (n = at least four samples each experimental time). The blood was immediately centrifuged at 4000 rpm for 15 min at 4 °C, plasma collected, divided into aliquots and stored at −80 °C until assayed. Lung specimens were also collected at 2, 4 and 6 h after antibiotic treatments to evaluate antibiotic tissue penetration into the lung. Finally, urinary samples were collected from the metabolic cage after the 6 h interval in order to measure CTX elimination.

The CTX concentrations were determined in triplicate by a validated large-plate agar diffusion technique, according to Good Laboratory Practice (GPL) standards, using the Mueller–Hinton Agar (Oxoid, UK) as the culture medium and *Escherichia coli* K12 as the test organism, with a lower limit of sensitivity of 0.125 mg/L. Standard concentrations were prepared daily in pooled plasma for plasma samples, in saline for the urine and lung samples. The test organism (1 × 10^6^ CFU/mL) was added using the surface layer technique. After homogeneous distribution of the culture, the excess liquid was removed with a pipette. The plates were incubated at 37 °C in air overnight. Best-fit standard curves were obtained by linear regression analysis. The correlation coefficient was no less than 0.99. For all CTX samples, intra-assay precision ranged from 1.5 to 6.8% and the inter-assay precision at a level of 0.75 mg/L ranged from 4.6 to 5.6%. Amicon R 10 K filters (Sigma Aldrich, Milan Italy) were used for the determination of free drug concentration (the filter cut-off being 10.000 nominal molecular weight limit). In short, 0.5 mL of plasma were stratified on the filters and centrifuged at 4000*g* for 30 min. The filtrate was collected and analyzed with the same bioassay described above.

Diuresis was collected for each rat (at least four rats each group). The total amount of diuresis in the 0–6 h interval of time was noted. Urine was stored at −20 °C until examination.

Changes of GFB-associated glycocalyx, in particular in the sialic components, was performed by lectin histo-chemistry. At the end of the experimental time, after the pentobarbital overdose, a midline incision was made in the abdomen, and kidney specimens were fixed in Carnoy’s fluid and routinely processed to obtain 6 μm-thick paraffin sections. *Maackia amurensis* agglutinin (MAA) and *Sambucus nigra* agglutinin (SNA) digoxigenin (DIG) labeled lectins (Roche Diagnostic, Mannheim, Germany) were used to identify sialic acids linked α2–3 and α2–6 to galactose or galactosamine, respectively. Lectin histo-chemistry was performed as previously described [16, 17]. In short, sections were treated with 20% acetic acid to inhibit the endogenous alkaline phosphatase, then treated with 10% blocking reagent in Total Buffered Saline (TBS) to reduce the background labelling. Afterwards, sections were washed in TBS and rinsed in Buffer 1, then incubated in DIG-labelled lectins diluted in Buffer 1 (1 and 5 μL/mL for SNA and MAA respectively) for 1 h at room temperature. Sections were then rinsed in TBS, incubated with anti-digoxigenin, conjugated with alkaline phosphatase diluted in TBS and washed in TBS. Labelling of the sites containing bound lectin-digoxigenin was obtained by incubating slides with Buffer 2 containing nitroblue tetrazolium (NBT)/X-phosphate (Roche Diagnostics, Mannheim, Germany) [16, 18].

Controls for lectin specificity included pre-incubation of lectins with the corresponding hapten sugars [16, 18]. For evaluation of the lectin-stained location, ten random fields (40× ocular) in each section (five sections for each specimen) were examined using light microscopy. A densitometric analysis was also carried out for lectin reactivity intensity by measuring the average optical density (OD) of region of interest (ROI, 40 µm^2^ area), using ImageJ National Institute of Health (NIH, Bethesda, MD, USA) software. Measured values were normalized to background [(OD-ODbkg)/ODbkg]. At least eight regions of interest in ten different optical fields were analyzed in each experiment.

For the PD analysis, the CTX concentration data on plasma were used to develop a population model by non- linear mixed effects modelling (using SAS 9.3 PROC NLMIXED).

We considered the following first-order compartment model for these

$$C_{it} = \frac{{Dk_{ei} k_{ai} }}{{Cl_{i} \left( {k_{ai} - k_{ei} } \right)}} \left[ {e^{{ - k_{ei} t}} - e^{{ - k_{ai} t}} } \right] + e_{it}$$

where *C*_*it*_ is the observed concentration of the i-th subject at time t, *D* is the dose of ceftriaxone, *k*_*ei*_ is the elimination rate constant for subject *i*, *k*_*ai*_ is the absorption rate constant for subject *i*, *Cl*_*i*_ is the clearance for subject *i*, and *e*_*it*_ are normal errors. To allow for random variability between subjects, we assumed:

$$Cl_{i} = e^{{\beta_{1} + b_{i1} }}$$

$$k_{ei} = e^{{\beta_{2} + b_{i2} }}$$

$$k_{ai} = e^{{\beta_{3} }}$$

where the β_s_ denote fixed-effects parameters and the b_is_ denote random-effects parameters with an unknown covariance matrix.

To obtain effect estimation and random effect joint distribution the matrix dual quasi-Newton algorithm was used in order to achieve convergence.

Two dosing regimens (100 and 200 mg/kg, corresponding to 1 and 2 g in humans respectively [15]) were simulated using Monte Carlo simulation based on the PK model. Each simulation generated concentration–time profiles from 10,000 rats per dosage regimen. Based on this data, T_free_ > MIC and the probability target attainment (PTA) for different multiples of MIC, were estimated graphically. MIC values according to the EUCAST breakpoints were considered, in which CTX susceptibility for E*nterobacteriaceae* was ≤1 mg/L.

The free concentrations (C_free_) were estimated from the total concentrations (*C*_*tot*_) using in vivo binding parameters of CTX and the following equation described by Kodama et al. [19]:

$$C_{{free}} = \frac{1}{2}\left[\begin{gathered}- \left( {nP + ~\frac{1}{{k_{{aff}} }} - ~C_{{tot}} } \right) \hfill \\ + ~\sqrt {\left( {nP + ~\frac{1}{{k_{{aff}} }} - ~C_{{tot}} } \right)^{2} + \frac{{4C_{{tot}} }}{{K_{{aff}} }}} \hfill \end{gathered} \right]$$

The following binding of CTX was used: the total concentration of protein binding sites (nP) 517 μmol l^−1^ and the binding affinity constant (K_aff_) 0.0367 l μmol^−1.^

In order to assess differences in continuous variables between the sham and CLP groups the *t* test or the Mann–Whitney test were used. The test choice was made following the Shapiro–Wilk test result for normality distribution of the variable in each group.

The difference between sham and CLP groups in PK parameters was assessed by Welch’s t-test. Statistical significance was set at p value lower than 0.05.

## Results

Sepsis was induced by CLP as documented by clinical signs (lethargy, pilo-erection, tachypnea) and growth of microorganisms in the peritoneal cavity. No spontaneous deaths occurred during the 6 h period. Both Gram-positive and Gram-negative strains (*Escherichia coli* 70%, *Enterococcus faecalis* 40%, *Streptococcus viridans* 15%, and coagulase-negative *Staphylococci* 70%) were collected at 6 h following surgery. Colony-forming units/mm^3^ ranged from ≤1 × 10^1^ in sham-operated rats to 5 × 10^4^–1 × 10^5^ in CLP rats.

**Fig. 2:**
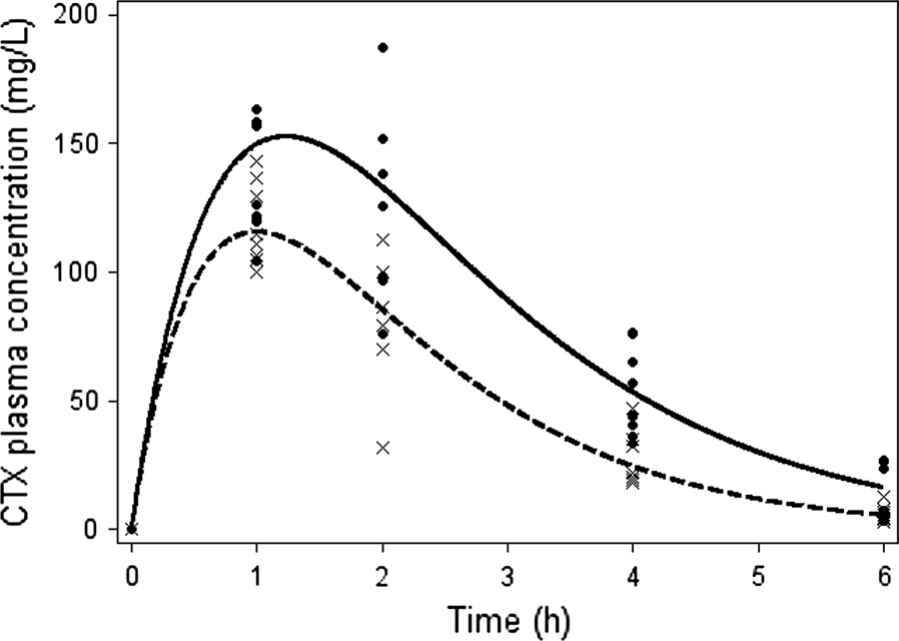
Total CTX plasma concentration (mg/L) time curve in septic (CLP, ●) and control (sham–operated, ×) rats (n = at least four rats each point)

The kinetic plasma profile of CTX in both groups is reported in Fig. 2. Peak plasma values were 132,48 and 120,29 mg/L respectively in the CLP and sham-operated control groups. Plasma levels remained higher in septic than in sham-operated rats, with a statistical significance between the 2nd and 4th h (difference at 2 h 47.3 mg/L p = 0.012; difference at 4 h 24.94, p = 0.004). Clearance was significantly lower in CLP rats (0.21 vs 0.32 mL/min p = 0.009). Despite the higher plasma levels, free/unbound CTX penetration tended to be lower in lung tissue in septic rats than in controls (lung/plasma value being at 2 h, p = 0.2; at 4 h, p = 0.05; at 6 h, p = 0.6). (see Table 1) In septic rats, the calculated AUC lung/AUC free plasma concentration ratio was 0.33 while in the sham it was 0.51.

**Table 1:** Free Ctx Lung Concentrations (mean ± SD) in sham and CLP rats

Hours	SHAM + CTX (mg/L)	CLP + CTX (mg/L)
2	5.7 ± 0.6	5.0 ± 0.7
4	2.7 ± 1.2	2.6 ± 0.7
6	0.4 ± 1.7	1.0 ± 0.2

**Fig. 3:**
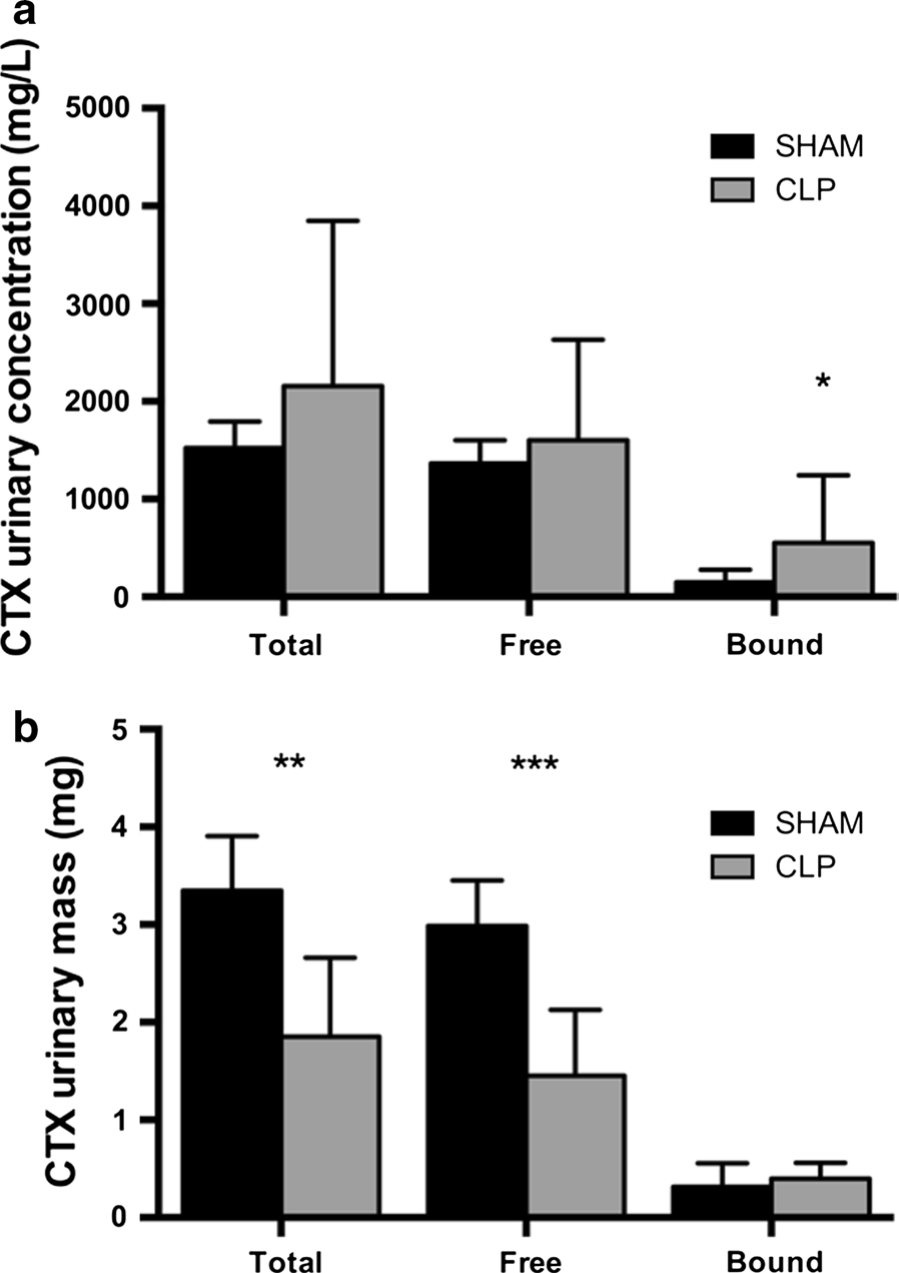
**a** CTX urinary concentration (mg/L) at the end of the experimental time (6 h). A significant increased quantity of bound-CTX was present in the urine of septic rats (553.53 vs 149.27 mg/L; *p < 0.05 CLP vs SHAM). **b** Total urinary loss of CTX (mg) at the end of the experimental time (6 h); the quantity was the product of the 6 h urinary volume and CTX concentration. Due to a reduced urinary output, CTX total loss was reduced in septic rats (1.85 vs 3.34 mg *p < 0.05)

The concentration of bound CTX in the urine was significantly higher in septic CLP-treated rats than in sham rats (553 ± 689 vs 149 ± 128 mg/L, p < 0.05; % of bound/total CTX 22 ± 6 vs 11 ± 4, p < 0.01 respectively in CLP and sham rats). However the total amount of CTX eliminated in the urine (calculated as urinary volume X CTX urinary concentration) was higher in sham than in CLP rats (3.34 ± 0.55 vs 1.85 ± 0.8 mg, p < 0.05) because urinary volume in the time interval 0-6 h was higher in sham than in septic rats (2.25 ± 0.53 vs 1.24 ± 0.72 mL, p < 0.05). For details see Fig. 3a, b.

**Fig. 4:**
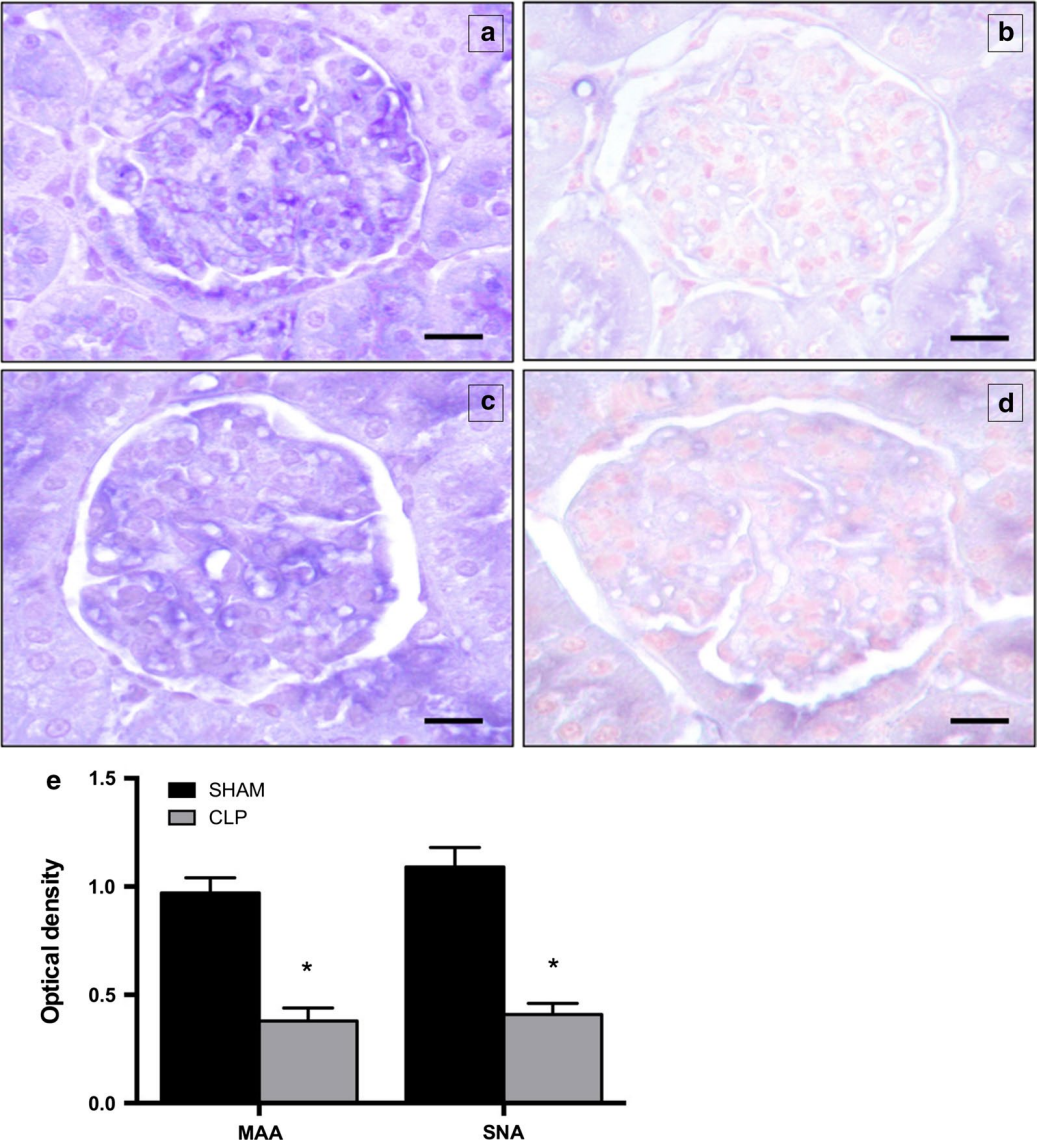
Representative light microphotograph of *Maackia amurensis* agglutinin (MAA) (**a, b**) and *Sambucus nigra* agglutinin (SNA) (**c**,** d**) reactivity at 6 h in sham-operated and CLP-treated rats. Lectin reactivity is present in the glomerular barrier of both study groups; however, reactivity in glomerulus of CLP rats (**b**,** d**) appears weaker than in sham-operated ones (**a**, **c**). **e** The quantitative analysis for SNA and MAA content showed that MAA and SNA reactivity intensity is significantly lower in CLP compared to sham-operated samples (MAA: 0.28 vs 0.97; SNA 0.41 vs 1.09 *p < 0.01 CLP vs SHAM). *Scale bar* = 25 mm. Data are the mean of OD values, at 6 h, both for sham-operated and CLP rats

The integrity of the GFB-associated glycocalyx was assessed by lectin histo-chemistry analysis. MAA and SNA reactivity (showing the presence of sialic acids linked α-2,3 and α-2,6 to galactose/galactosamine, respectively) was significantly lower in the glomerular barrier in the CLP rats with respect to the sham-operated rats (MAA 0.38 ± 0.06 vs 0.97 ± 0.07, p < 0.01; SNA 0.41 ± 0.05 vs 1.09 ± 0.09, p < 0.01, Fig. 4).

Monte Carlo simulation was performed to evaluate the PTA attainment of T_free_ > MIC for over 80% and for 100% of the dosing interval.

**Fig. 5:**
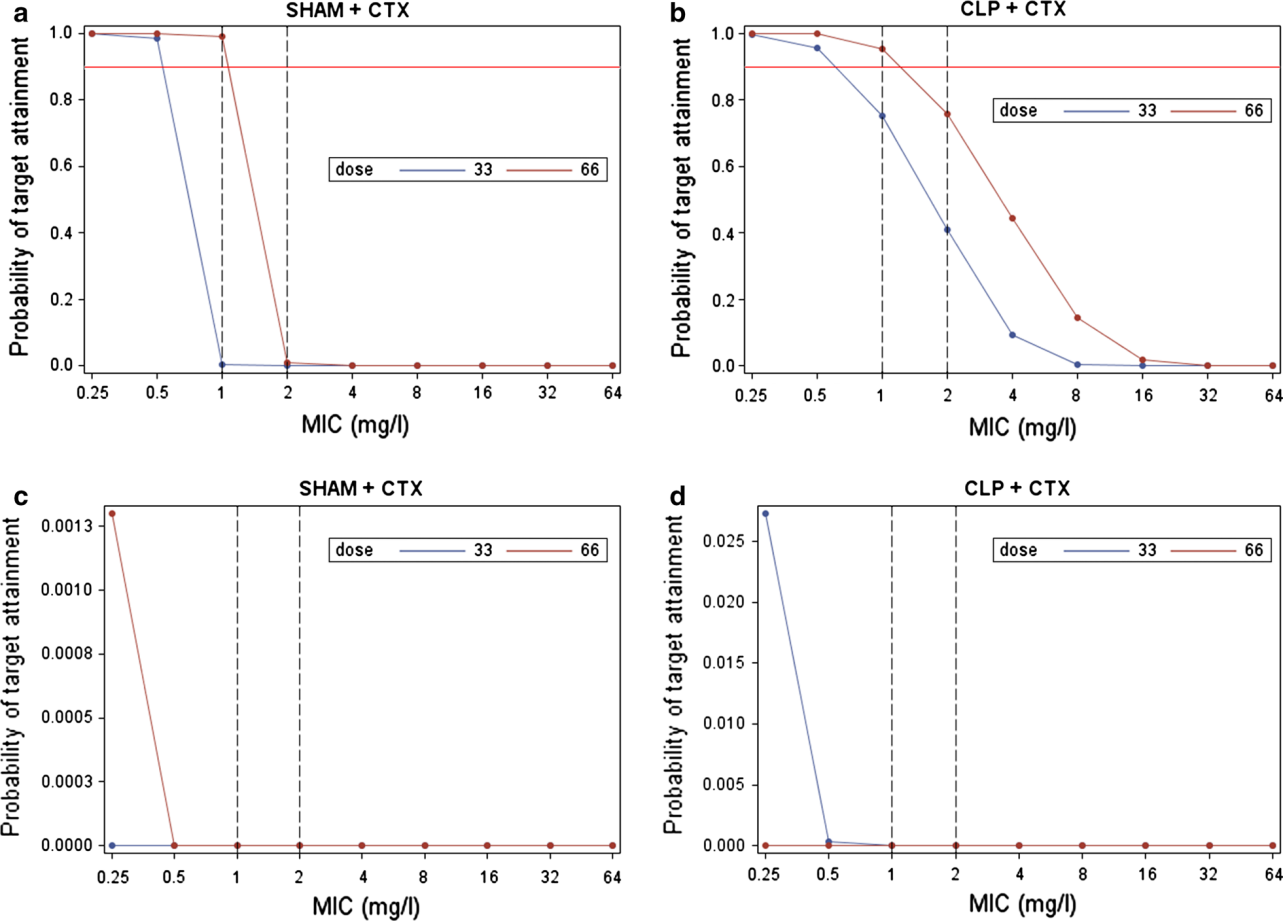
Monte Carlo simulation was performed to evaluate the PTA attainment of T_free_ > MIC for over 80% and for 100% of the dosing interval. The PTA for the 80% dosing interval was 0% for MIC = 1 mg/L with the 1 g dose, and 99% with the 2 g dose in the sham rats. In the CLP rats, the PTA for the 80% dosing interval was 75% for MIC = 1 for the 1 g dose and 95% for the 2 g dose (**a**, **b**). Probability of target attainment (PTA) was calculated for the two dosing regimens 100 (*blue line*) and 200 (*red line*) mg/kg (corresponding to 1 and 2 g in humans respectively), using Monte Carlo simulation based on the PK model. The time period during which free concentrations remained higher then MIC (T_free_ > MIC) and the PTA for different multiples of MIC were estimated graphically. MIC values according to the EUCAST breakpoints were considered, with CTX susceptibility for E*nterobacteriaceae* being ≤1 mg/L [34]. The PTA for the 100% dosing interval was 0% for MIC = 1 mg/L for both the 1 g and 2 g doses in both the sham rats (**c**) and the CLP rats (**d**)

The PTA for the 80% dosing interval was 0% for MIC = 1 mg/L with the 1 g dose, and 99% with the 2 g dose in the sham rats. In the CLP rats, the PTA for the 80% dosing interval was 75% for MIC = 1 for the 1 g dose and 95% for the 2 g dose (Fig. 5a, b).

The PTA for the 100% dosing interval was 0% for MIC = 1 mg/L for both the 1 g and 2 g doses in both the sham rats (Fig. 5c) and the CLP rats (Fig. 5d).

## Discussion

Our data show that the PK of CTX is modified from the very initial phases of sepsis in the sense that plasma concentrations are higher but lung penetration is generally lower compared to non-septic sham controls. Another important finding in our study is that, due to sepsis-induced changes in GFB perm-selectivity, urinary loss of bound CTX is higher in septic than in sham rats. Finally, according to Monte Carlo simulation, despite the higher plasma levels of total CTX in septic rats, the dose administered which is equivalent to 1 g in humans is insufficient to reach a PTA >90% both for 80 and 100% of the dosing interval.

It is well known that optimal antimicrobial therapy is characterized by a PK/PD and time-targeted protocol in order to improve patients’ outcome. Therefore we have studied sepsis in its early phase to assess its effects concerning the initial optimization of antimicrobial therapy. We have used the same model which we used in the past [12, 16] since it is suitable for the study of the first hours of sepsis because cytokine activation, microorganism growth, impairment of the integrity of the sialic components of the GFB with consequent albuminuria have already occurred within this period of time [6, 18]. In the present study we have expanded our observations on early sepsis by examining whether modifications of highly-bound molecules like CTX were present and whether they were associated with increased renal loss of CTX.

We chose to study CTX because, although it is an “old” cephalosporin, it is commonly prescribed, being the second most popular in acute care hospitals in the USA [20, 21]. In some countries, it is also available in combination with the Beta-lactamases inhibitor Sulbactam and in this case it maintains a role for the treatment of ESBL-positive strains [22]. Moreover, it is interesting to evaluate the effects of sepsis and of increased GFB permeability on a drug which is extensively bound (>95%) and whose renal elimination occurs almost exclusively throughout glomerular filtration [23].

For all cephalosporins, the T_free_ > MIC of the dosing interval is the PK/PD parameter which best predicts their efficacy, and the T_free_ > MIC of 80–100% of the dosing interval for CTX has been considered the optimal target [9].

In a study on the PK-PD of CTX in 54 critically ill septic patients [24], the CTX PK parameters were extremely variable: half–life ranged between 0.8 and 28 h, and the clearance from 4 to 33 mL/min (=0.26–1.98 L/h), due to the different types of patients and/or organ failure. Despite this variability and no stratification, the authors conclude that with a 2 g dose CTX plasma levels are sufficient to exert maximal antimicrobial activity against all the most common susceptible abdominal pathogens. Garot et al. [8] who performed a Monte Carlo simulation on similar patients, showed that even with a 1 g dose a PTA >90 of 100% of the dosing interval can be achieved for MICs ≤1 mg/L and for Cl ≤120 mL/min. On the contrary, on the basis of our Monte Carlo simulation in our experimental model, even with the 2 g dose the optimal PK/PD target could not be guaranteed in the early phases of sepsis.

Acute Kidney Injury (AKI) is frequently observed in ICU patients but in the early phases of sepsis an increase in creatinine clearance has been described as well. A GFR above 130 mL/1.73 m^2^ has been found to be associated with sub-therapeutic trough concentrations of beta-lactams [25]. However, so far the permeability properties of the GFB has not received attention [18]. Permeability of the glomerulus is indeed a highly specialized function which limits the passage of proteins and larger molecules but allows water and small molecules to cross. This “perm-selectivity” is guaranteed only when the structural and ultra-structural integrity of the GFB is maintained, including that of its endothelium-associated glycocalyx, which is composed of membrane-bound glycoproteins and proteoglycans [26, 27].

In our experimental conditions, we have found that the loss of the sialic component of glycocalyx was associated with higher CTX-bound urinary loss in septic rats, thus supporting the hypothesis of a loss of perm-selectivity (16).

Antibiotic penetration into the lung is critical in order to obtain good clinical outcomes in ICU patients with pneumonia. Lung penetration of CTX should be theoretically similar to the percentage of the quantity of free drug and it should have been higher for septic rats than for controls. However, tissue penetration of betalactams depends not only on linear plasma concentrations [28] but also on other factors, such as their physico-chemical properties, changes in membrane permeability, re-arrangement of pulmonary circulation [29] and the tissue alteration due to the action of free-radical oxygen production [30]. Therefore, not surprisingly, we observed reduced penetration in septic than in control lungs, in accordance with other studies [29, 31]. The higher plasma levels we found in septic rats may have several causes, such as higher intestinal absorption due to intestinal inflammatory vaso-dilation and reduced penetration into peripheral tissues. Finally, total CTX renal elimination depends not only on the CTX concentration but also on urinary volume and in our study septic rats produced less urine.

## Limits of the study

This was a PK/PD study carried out on the rat, therefore its extrapolation to humans can only be hypothetical. However, the dose of 100 mg/kg we have used gives peak plasma levels similar to those observed in humans after 1 g iv administration and the experimental time of 6 h may be equivalent to the 24 h interval in humans according to trough values [15]. Significantly, the level of protein binding is similar in humans and rats [32], thus contributing to support the validity of our results: it underlines therefore the importance of taking into consideration the loss of bound antimicrobials through urine during sepsis. Other limitations of our study are: (1) the fact that we have measured CTX lung penetration in the homogenate of lung tissue and not in the Epithelial Lining Fluid; (2) we do not know if the administration of fluids would have increased urinary volume and thus the total loss of CTX. Finally, a 24 h circadian rhythm in CTX clearance has been shown which might contribute to daily CTX level variations; this is important especially for drugs administered once a day [33]. However, all the experiments were conducted in the same time-frame (starting in the morning at 9 am).

## Conclusions

A rapid attainment of therapeutic targets of antimicrobials is a crucial element for an appropriate antimicrobial therapy in critically ill patients [10]. Our study confirms how sepsis impacts on the PK of CTX since the initial phase. Moreover it shows, for the first time, that among factors that can affect drug pharmacokinetics during the early phases of sepsis, urinary loss of both free and albumin-bound antimicrobials should be considered.

In view of the difficulty of predicting sepsis-associated changes in CTX PK, and in order to preserve the activity of this “old” antimicrobial and reduce the risk of resistance, the best way to adjust the dosage of CTX in critically ill patients appears to be therapeutic drug monitoring. Further studies are necessary to confirm these results in humans.

## Abbreviations

GFB: glomerular filtration barrier
PK/PD: pharmacokinetics–pharmacodynamics
CTX: ceftriaxone
CLP: cecal ligation and puncture
Vd: volume of distribution
ICU: intensive care unit
CrCL: creatinine clearance
MAA: *Maackia amurensis* agglutinin
SNA: *Sambucus nigra* agglutinin
DIG: digoxigenin
PTA: probability target attainment
C_free_: free concentrations
C_tot_: total concentrations
MIC: minimum inhibitory concentration
AKI: acute kidney injury
CFU: colony forming unit
HPLC: high performance liquid chromatography
GPL: good laboratory practice
TBS: total buffered saline
AUC: area under the curve
ESBL: extended-spectrum beta-lactamase
i.p.: intra-peritoneally

## References

[1] Blot SI, Pea F, Lipman J (2014). The effect of pathophysiology on pharmacokinetics in the critically ill patient–concepts appraised by the example of antimicrobial agents. Adv Drug Deliv Rev.

[2] Sime FB, Udy AA, Roberts JA (2015). Augmented renal clearance in critically ill patients: etiology, definition and implications for beta-lactam dose optimization. Curr Opin Pharmacol.

[3] Pea F (2013). Plasma pharmacokinetics of antimicrobial agents in critically ill patients. Curr Clin Pharmacol.

[4] Udy AA, Roberts JA, De Waele JJ, Paterson DL, Lipman J (2012). What’s behind the failure of emerging antibiotics in the critically ill? Understanding the impact of altered pharmacokinetics and augmented renal clearance. Int J Antimicrob Agents.

[5] Chelazzi C, Villa G, Mancinelli P, De Gaudio AR, Adembri C (2015). Glycocalyx and sepsis-induced alterations in vascular permeability. Crit Care.

[6] De Gaudio AR, Adembri C, Grechi S, Novelli GP (2000). Microalbuminuria as an early index of impairment of glomerular permeability in postoperative septic patients. Intensive Care Med.

[7] Goodman LS, Gilman AG (2011). Goodman and Gilman’s the pharmacological basis of therapeutics.

[8] Garot D, Respaud R, Lanotte P, Simon N, Mercier E, Ehrmann S (2011). Population pharmacokinetics of ceftriaxone in critically ill septic patients: a reappraisal. Br J Clin Pharmacol.

[9] Perry TR, Schentag JJ (2001). Clinical use of ceftriaxone: a pharmacokinetic–pharmacodynamic perspective on the impact of minimum inhibitory concentration and serum protein binding. Clin Pharmacokinet.

[10] Kollef MH, Sherman G, Ward S, Fraser VJ (1999). Inadequate antimicrobial treatment of infections: a risk factor for hospital mortality among critically ill patients. Chest.

[11] Schleibinger M, Steinbach CL, Töpper C, Kratzer A, Liebchen U, Kees F (2015). Protein binding characteristics and pharmacokinetics of ceftriaxone in intensive care unit patients. Br J Clin Pharmacol.

[12] Venturi L, Miranda M, Selmi V, Vitali L, Tani A, Margheri M (2009). Systemic sepsis exacerbates mild post-traumatic brain injury in the rat. J Neurotrauma.

[13] Hubbard WJ, Choudhry M, Schwacha MG, Kerby JD, Rue LW, Bland KI (2005). Cecal ligation and puncture. Shock..

[14] Dejager L, Pinheiro I, Dejonckheere E, Libert C (2011). Cecal ligation and puncture: the gold standard model for polymicrobial sepsis?. Trends Microbiol.

[15] B. Braun Medical Inc. Ceftriaxone and dextrose. 2015. http://www.drugs.com/pro/ceftriaxone-and-dextrose.html. Accessed 14 July 2016.

[16] Adembri C, Selmi V, Vitali L, Tani A, Margheri M, Loriga B (2014). Minocycline but not tigecycline is neuroprotective and reduces the neuroinflammatory response induced by the superimposition of sepsis upon traumatic brain injury. Crit Care Med.

[17] Marini M, Ambrosini S, Sarchielli E, Thyrion GDZ, Bonaccini L, Vannelli GB (2014). Expression of sialic acids in human adult skeletal muscle tissue. Acta Histochem.

[18] Adembri C, Sgambati E, Vitali L, Selmi V, Margheri M, Tani A (2011). Sepsis induces albuminuria and alterations in the glomerular filtration barrier: a morphofunctional study in the rat. Crit Care.

[19] Kodama Y, Kuranari M, Tsutsumi K, Kimoto H, Fujii I, Takeyama M (1992). Prediction of unbound serum valproic acid concentration by using in vivo binding parameters. Ther Drug Monit.

[20] Dumartin C, L’Hériteau F, Péfau M, Bertrand X, Jarno P, Boussat S (2010). Antibiotic use in 530 French hospitals: results from a surveillance network at hospital and ward levels in 2007. J Antimicrob Chemother.

[21] Magill SS, Edwards JR, Beldavs ZG, Dumyati G, Janelle SJ, Kainer M (2014). Prevalence of antimicrobial use in US acute care hospitals, May–September 2011. JAMA.

[22] Sharma VD, Singla A, Chaudhary M, Taneja M (2015). Population pharmacokinetics of fixed dose combination of ceftriaxone and sulbactam in healthy and infected subjects. AAPS Pharm Sci Tech..

[23] Patel IH, Chen S, Parsonnet M, Hackman MR, Brooks MA, Konikoff J (1981). Pharmacokinetics of ceftriaxone in humans. Antimicrob Agents Chemother.

[24] Joynt GM, Lipman J, Gomersall CD, Young RJ, Wong EL, Gin T (2001). The pharmacokinetics of once-daily dosing of ceftriaxone in critically ill patients. J Antimicrob Chemother.

[25] Udy AA, Varghese JM, Altukroni M, Briscoe S, McWhinney BC, Ungerer JP (2012). Subtherapeutic initial β-lactam concentrations in select critically ill patients: association between augmented renal clearance and low trough drug concentrations. Chest.

[26] Fu BM, Tarbell JM (2013). Mechano-sensing and transduction by endothelial surface glycocalyx: composition, structure, and function. Wiley Interdiscip Rev Syst Biol Med..

[27] Arkill KP, Neal CR, Mantell JM, Michel CC, Qvortrup K, Rostgaard J (2012). 3D reconstruction of the glycocalyx structure in mammalian capillaries using electron tomography. Microcirculation..

[28] Lodise TP, Butterfield J (2011). Use of pharmacodynamic principles to inform β-lactam dosing: “S” does not always mean success. J Hosp Med.

[29] Hutschala D, Kinstner C, Skhirtladze K, Mayer-Helm BX, Zeitlinger M, Wisser W (2008). The impact of perioperative atelectasis on antibiotic penetration into lung tissue: an in vivo microdialysis study. Intensive Care Med.

[30] Galvào AM, Wanderley MSO, Silva RA, Filho CAM, Melo-Junior MR, Silva LA (2014). Intratracheal co-administration of antioxidants and ceftriaxone reduces pulmonary injury and mortality rate in an experimental model of sepsis. Respirology.

[31] Lodise TP (2011). Penetration of meropenem into epithelial lining fluid of patients with ventilator-associated pneumonia. Antimicrob Agents Chemother.

[32] Craig WA, Lorian V (1991). Protein-binding and the antimicrobial effects: methods for the determination of protein-binding. Antibiotics in laboratory medicine.

[33] Rebuelto M, Ambros L, Rubio M (2003). Daily variations in ceftriaxone pharmacokinetics in rats. Antimicrob Agents Chemother.

[34] Committee TE, Testing AS, Changes N, Pseudomonas E. European Committee on Antimicrobial Susceptibility Testing Breakpoint tables for interpretation of MICs and zone diameters European Committee on Antimicrobial Susceptibility Testing Breakpoint tables for interpretation of MICs and zone diameters. http://www.eucast.org/fileadmin/src/media/PDFs/EUCAST_files/Breakpoint_tables/v_5.0_Breakpoint_Table_01.pdf. 2015;0–77. Available from: http://www.eucast.org/fileadmin/src/media/PDFs/EUCAST_files/Breakpoint_tables/v_5.0_Breakpoint_Table_01.pdf. Accessed 14 July 2016.

